# Glucocorticoid treatment and new‐onset hyperglycaemia and diabetes in people living with chronic obstructive pulmonary disease: A systematic review and meta‐analysis

**DOI:** 10.1111/dme.15475

**Published:** 2024-12-06

**Authors:** Rajna Golubic, Hudson Mumbole, Mouhamad Hussein Ismail, Alwyn Choo, Olivia Baker, Karyna Atha, Sarah Chew Sue Mei, Arjun Raj, Preethu Anand, Nwe Oo Aung, Niraj S. Kumar, Tulika Nahar, Ruth L. Coleman, Jeremy W. Tomlinson, Najib Rahman, Rishi Caleyachetty, Amanda Adler

**Affiliations:** ^1^ Diabetes Trials Unit, Oxford Centre for Diabetes, Endocrinology and Metabolism, and NIHR Oxford Biomedical Research Centre University of Oxford Oxford UK; ^2^ Addenbrooke's Hospital Cambridge University Hospitals NHS Foundation Trust Cambridge UK; ^3^ Norfolk and Norwich University Hospitals NHS Foundation Trust Norwich UK; ^4^ Harris Manchester College University of Oxford Oxford UK; ^5^ Leicester University Hospitals NHS Foundation Trust Leicester UK; ^6^ Stoke Mandeville Hospital Aylesbury UK; ^7^ Department of Cardiovascular Sciences University of Leicester Leicester UK; ^8^ National Medical Research Association London UK; ^9^ Queen's University Belfast Belfast UK; ^10^ Oxford Respiratory Trials Unit University of Oxford Oxford UK; ^11^ Warwick Medical School Health Sciences University of Warwick Warwick UK

**Keywords:** diabetes, clinical diabetes, other complications, systematic review

## Abstract

**Introduction:**

In people living with chronic obstructive pulmonary disease (COPD), we aimed to estimate: (1) the prevalence of glucocorticoid‐induced hyperglycaemia (GIH); (2) whether the prevalence of GIH varies by age, baseline diabetes status, treatment duration, ascertainment of glycaemia, definition of hyperglycaemia, study design and year of publication; and (3) the relative risk (RR) of new‐onset hyperglycaemia in exposed vs non‐exposed to systemic glucocorticoids.

**Methods:**

We searched electronic databases until 9 November 2023 for randomised controlled trials and observational studies including adults diagnosed with COPD, with or without diabetes at baseline, using systemic glucocorticoids equivalent to prednisolone ≥5 mg/day for ≥3 days if exposed. Hyperglycaemia was defined as a blood glucose above a study‐specific cut‐off. We extracted data on study and participant characteristics, exposure and outcome. We performed random‐effects meta‐analysis to calculate pooled prevalence estimate of GIH. Prevalence was expressed as the proportion of people who developed hyperglycaemia among all exposed to systemic glucocorticoids during follow‐up. We calculated RR of new‐onset hyperglycaemia in exposed vs non‐exposed to systemic glucocorticoids from eight studies.

**Results:**

Of 25,806 citations, we included 18 studies comprising 3642 people of whom 3125 received systemic glucocorticoids and 1189 developed hyperglycaemia. Pooled prevalence of GIH was 38.6% (95%CI 29.9%–47.9%) with significant heterogeneity, *I* ^2^ = 96% (*p* < 0.010), which was partially explained by differences in study design. Pooled RR = 2.39 (95%CI 1.51–3.78). Publication bias was present.

**Conclusion:**

The prevalence of GIH was 38.6%. Being treated with systemic glucocorticoids for COPD was associated with 2.4 times higher risk of new‐onset hyperglycaemia versus no glucocorticoid treatment.

**What's new?**

- The prevalence of glucocorticoid‐induced hyperglycaemia in people with COPD and the risk of new‐onset hyperglycaemia associated with glucocorticoid treatment has not been comprehensively quantified.
- Using data from 18 studies, we found that the prevalence of glucocorticoid‐induced hyperglycaemia was 38.6%. Treatment with systemic glucocorticoids for COPD was associated with 2.4 times higher risk of new‐onset hyperglycaemia versus no glucocorticoid treatment.
- Healthcare providers looking after people with COPD who are treated with systemic glucocorticoids should consider measuring blood glucose before starting systemic glucocorticoid therapy and throughout the treatment course to enable timely detection and management of glucocorticoid‐induced hyperglycaemia.

## 1 INTRODUCTION

Glucocorticoids are widely used to treat people across a range of conditions including inflammatory and autoimmune conditions, haematological disorders, malignancies and also to treat hospitalised people with COVID‐19.^1^ Glucocorticoids are associated with hyperglycaemia and new‐onset diabetes.^2^ Hyperglycaemia is independently associated with an increased risk of mortality and prolonged hospital stay.^3^

While the association between treatment with glucocorticoids and hyperglycaemia is well‐recognised, it is relatively understudied. Previous systematic reviews of glucocorticoid‐induced hyperglycaemia (GIH) include retrospective studies without comparison to the unexposed to glucocorticoids.^4^, ^5^, ^6^ Another systematic review^7^ included randomised controlled trials (RCTs), but focussed on only one indication for glucocorticoids (respiratory diseases). These studies demonstrated that exposure to exogenous systemic glucocorticoids increases the risk of new‐onset diabetes in those without known diabetes and the risk of hyperglycaemia in those with known diabetes.

According to the Global Burden of Disease 2019 Study,^8^ chronic obstructive pulmonary disease (COPD) is a major public health problem accounting for 212.3 million prevalent cases and 3.3 million deaths making it the third leading cause of death globally.^9^ COPD is also associated with substantial economic loss due to disability with 74.4 million Disability Adjusted Life Years (DALYs) in 2019.^8^ Acute exacerbations of COPD are treated with systemic glucocorticoids among other therapeutic modalities. Previously published systematic review focussed on the risk of GIH in people with respiratory diseases^7^ and included a variety of respiratory diseases analysed together rather than COPD alone and the percentage of people with COPD could not be calculated. Therefore, the magnitude of this risk has not been comprehensively quantified in people living with COPD.

The objectives of this systematic review were to estimate the following in people living with COPD:

1. The prevalence of GIH and glucocorticoid‐induced diabetes (GID);
2. Whether the prevalence of GIH varies according to age, diabetes status at baseline, treatment duration, method of ascertainment of glycaemia, definition of hyperglycaemia, study design and year of publication; and
3. The relative risk (RR) of new‐onset hyperglycaemia in people exposed vs non‐exposed to systemic glucocorticoids

## 2 METHODS

We conducted this study according to the Preferred Reporting Items for Systematic Reviews and Meta‐Analyses (PRISMA) guidelines.^10^ The protocol is registered on PROSPERO (CRD482601).

### 2.1 Search strategy

We searched PubMed, EMBASE, Cochrane Library and ClinicalTrials.gov from inception to 9 November 2023. The search strategies are shown in Table S1. We applied search filters related to adult humans, and used no restrictions for language, year of publication or indications for glucocorticoid use. We supplemented the search by reviewing reference lists of relevant articles.

### 2.2 Study selection

We used the following criteria:

*Population*: adults (aged≥18 years) diagnosed with COPD, with or without documented diabetes at study entry (i.e. information on history of diabetes).

*Exposure*: Systemic use of glucocorticoids (oral, intravenous or intramuscular) including dexamethasone, prednisone, prednisolone, methylprednisolone, hydrocortisone, budesonide, cortisone, deflazacort, plenadren and triamcinolone equivalent to prednisolone 5 mg/day (Table S2) or higher for ≥3 days with or without concomitant use of topical glucocorticoids. We chose prednisolone at 5 mg/day as it is equivalent to daily physiologic glucocorticoid production, and higher doses are associated with increased risk of adverse events including hyperglycaemia and adrenal insufficiency.^11^

*Study design*: RCTs, observational analyses of RCTs (GIH/GID as a safety outcome), cohort studies (prospective and retrospective), case–control studies and cross‐sectional studies.

*Publication*: Peer‐reviewed articles in academic journals, any language, any year of publication.

*We excluded studies*: conducted in children (age <18 years), limited to topical administration of glucocorticoids; systemic glucocorticoid therapy administered for ≤3 days; study design not listed above; or if a publication was not peer‐reviewed.

We exported the retrieved records from each database to Covidence,^12^ removed duplicate records and two reviewers (RG, MHI, AC, OB, KA, SCSM, AR, PA, NOA, NK, TN) independently performed title and abstract screening^12^ identifying potentially eligible studies. The same reviewers independently reviewed full texts of these articles and assessed them against eligibility criteria. RG and HM resolved disagreements.

### 2.3 Data extraction

We used a pre‐defined data extraction form to extract data (Table 3). This details variable type, description, classification and study details, population characteristics, exposure and outcome.

We extracted data on the first author's name, year of publication, study location, sample size, population age, sex distribution, body mass index (BMI), baseline diabetes status and details about COPD exacerbation and maintenance therapy if available. We collected data on duration of follow‐up, study design and for RCTs details about randomisation, blinding and interventions in each arm. We extracted exposure data including glucocorticoid name,^15^ dose, frequency, route of administration and duration of treatment, whether or not the reference category from that study contained only participants not exposed to glucocorticoids, and description of tapering regimen if available. Outcomes of interest were hyperglycaemia (defined as new‐onset hyperglycaemia in people with or without diabetes before starting glucocorticoid treatment) and diabetes (defined as new‐onset diabetes in people without prior diagnosis of diabetes). Outcome‐related data included the following: number and percentage of participants with new hyperglycaemia or diabetes (in each study); method of diagnosing new‐onset hyperglycaemia or diabetes; new glucose‐lowering therapy in response to hyperglycaemia; and type of glucose‐lowering treatment. If the prevalence or effect measures were not reported, we calculated prevalence based on the number of people who developed new‐onset hyperglycaemia or diabetes among those exposed to glucocorticoids, and the effect measure based on the number of people who did and did not develop new‐onset hyperglycaemia or diabetes among exposed and non‐exposed.

We tested the feasibility of the data extraction form in a randomly selected sample of articles. Six authors (MHI, AC, OB, SCSM, NK and TN) contributed to the data extraction and data from every included article were extracted by two independent authors. RG and HM resolved the discrepancies.

### 2.4 Risk of bias

We used the Cochrane Risk of Bias (RoB2) tool^16^ for RCTs and Newcastle–Ottawa quality assessment scale (NOS)^14^ for observational studies. NOS rates the quality of observational studies in meta‐analyses based on three domains: selection, comparability of groups and ascertainment of exposure and outcome.^14^ Two independent reviewers performed risk of bias assessment for observational studies (MHI, SCSM) and two independent reviewers assessed the risk of bias in RCTs (RG, HM).

### 2.5 Data synthesis

A minimum of three studies were required to conduct meta‐analysis; otherwise, we performed a narrative synthesis.

#### 2.5.1 Aim 1: To quantify the prevalence of GIH and GID

We expected high heterogeneity in the prevalence of GIH because of differences in pharmacokinetics of glucocorticoids and diabetes status at baseline. Therefore, we conducted random‐effects meta‐analysis (DerSimonian‐Laird method^17^) to calculate pooled estimates of the study‐specific prevalence estimates. We reported prevalence as a proportion of people with GIH or GID out of all exposed people in the study during follow‐up. This estimate approximates cumulative incidence, that is the number of new cases of hyperglycaemia or diabetes during at‐risk period.^18^ In four RCTs, systemic glucocorticoids were used in ≥2 arms^14^, ^16^, ^19^, ^20^; in these studies, we calculated prevalence by dividing the number of people with GIH or GID in all arms exposed to systemic glucocorticoids by the number of all exposed people. We analysed pooled prevalence in all included studies and then fitted separate random‐effects models using only studies that included people with and those without diabetes and baseline and studies that included only people without diabetes at baseline. We performed quantitative synthesis for GIH. Since only two studies reported GID, we used a narrative synthesis to summarise these findings.

#### 2.5.2 Aim 2 To estimate whether the prevalence of GIH in adults living with COPD varies according to age, diabetes status at baseline, treatment duration, method of ascertainment of glycaemia, definition of hyperglycaemia, study design and year of publication

We assessed the heterogeneity of prevalence estimates between studies using *I* ^2^‐statistic. The *I* ^2^‐statistic estimates the percentage of variance accounted for by a true difference rather than chance.^21^ Typically, *I* ^2^ values greater than 60%–70% indicate substantial heterogeneity. Sources of heterogeneity were explored further using subgroup analyses as detailed below.

The models with prevalence of GIH were stratified by age (< or ≥median (69 years)), diabetes status at baseline (people with or without diabetes, people without diabetes), treatment duration (<1 week, ≥1 week), study design (observational, interventional), method of ascertainment of glycaemia (venous blood glucose, plasma or serum glucose, not reported), definition of hyperglycaemia (blood glucose≥10 mmol/L, blood glucose≥11.1 mmol/L, other, not reported) and year of publication (before 2010, after 2010). We analysed subgroups by: (1) computing the pooled prevalence within each subgroup, and (2) comparing the pooled prevalence across the subgroups. Tests for heterogeneity were conducted across the subgroups.

#### 2.5.3 Aim 3: Among people with COPD, to quantify the RR of new‐onset hyperglycaemia in people exposed to systemic glucocorticoids compared to non‐exposed

We used RR to represent the prevalence ratio.^22^ If the study did not report RR of new‐onset diabetes or hyperglycaemia, we calculated RR according to the following formula where *a* represents the number of people who developed new‐onset hyperglycaemia or diabetes among those exposed to glucocorticoids, *b* represents the number of people who received glucocorticoids but did not develop new‐onset hyperglycaemia or diabetes, *c* represents the number of people who developed new‐onset hyperglycaemia or diabetes among those non‐exposed to glucocorticoids, and *d* represents the number of people who were not exposed to glucocorticoids and did not develop new‐onset hyperglycaemia or diabetes.^20^

$$\mathrm{RR}=\left[a/\left(a+b\right)\right]/\left[c/\left(c+d\right)\right]$$

In selected articles, information was available to calculate RR for new‐onset hyperglycaemia, but not for diabetes. We expected high heterogeneity in RR of GIH because of differences in pharmacokinetics of glucocorticoids and diabetes status at baseline and performed a random‐effects meta‐analysis (^17^) to calculate pooled estimates of the study‐specific RR. We defined the reference category as non‐exposure to systemic glucocorticoids. If the 95% CI for effect measures were not reported, but the point estimates and the p‐values were, we calculated 95% CI by^23^:

1. Calculating the log RR: lnRR = ln(RR)
2. Calculating the test statistic for a normal distribution test, *z*, from the reported *p*‐value^23^:

$$z=-0.862+\surd \left[0.743–2.404\times \ln\left(p\right)\right].$$

3 Calculating the standard error: [SE = lnRR/*z*]
4 Calculating 95% CI on the log‐scale:

95%CI:lnRR−1.96×SEto lnRR+1.96×SE

5 Calculating 95% CI on the original scale:

$$95\%\mathrm{CI}:\exp.\left(\text{lnRR}-1.96\times \mathrm{SE}\right)\;\text{to}\;\exp.\left(\text{lnRR}+1.96\times \mathrm{SE}\right).$$

We did not conduct meta‐regression because fewer than 10 studies in the model with RR were available.^24^

We performed sensitivity analyses excluding the studies with some concerns about bias identified by the RoB2 tool^25^ for RCTs and NOS^26^ for observational studies. We used Egger's test and funnel plots to examine publication bias.^27^, ^28^ All tests were two‐sided and *p* < 0.05 was considered statistically significant. We analysed data using STATA^29^ version 17 (Stata Corp, College Station, Texas, USA) and R^30^ version 4.4.0 (R Foundation for Statistical Computing, Vienna, Austria).

## 3 RESULTS

### 3.1 Study selection

**FIGURE 1:**
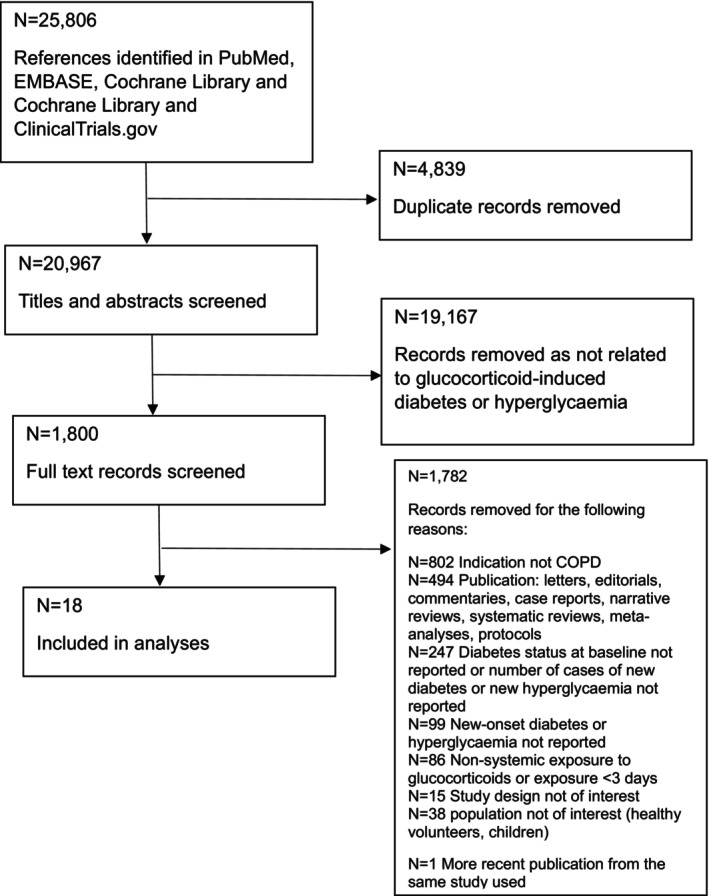
PRISMA flow diagram of the study.

The search identified *N* = 25,806 hits (Figure 1). After removing duplicates and screening titles and abstracts, we selected 1800 articles for full‐text review. We identified 18 articles^13^, ^14^, ^16^, ^19^, ^20^, ^31^, ^32^, ^33^, ^34^, ^35^, ^36^, ^37^, ^38^, ^39^, ^40^, ^41^, ^42^, ^43^ to include. The most common reasons to exclude studies were as follows: indication for glucocorticoids other than COPD, or publication type being letters, editorials, commentaries, case reports, narrative reviews, systematic reviews, meta‐analyses, or protocols.

### 3.2 Study characteristics

The characteristics of the 18 included studies are summarised (Table 1). In total, 3125 of 3642 included people were exposed to systemic glucocorticoids and 1189 of them developed new‐onset hyperglycaemia. Five hundred and seventeen of 3642 people were non‐exposed to glucocorticoids of whom 96 developed new‐onset hyperglycaemia. Two studies^13^, ^14^ recorded new‐onset diabetes and included a total of 382 people with 17 developing new‐onset diabetes.

**TABLE 1:** Characteristics of included studies.

First author (year of publication)	Study design	Region	Total *N* at baseline	Follow‐up duration (unit of time)	Age at baseline (years), mean (SD) or median (IQR)	Sex, *N* (%) female	BMI (kg/m^2^), mean (SD) or median (IQR)	*N* (%) exposed to glucocorticoids	*N* (%) non‐exposed to glucocorticoids	*N* (%) with diabetes at baseline	Systemic glucocorticoid regimen
Upadhyay et al.^13^	Retrospective cohort	North America	64	12 months	71 (13)	37 (58%)	Reported as: > 25 versus <25 kg/m^2^ Proportion of people with BMI > 25 25 kg/m^2^ 12/22 in normoglycaemic group and 24/42 in hyperglycaemic group	64 (100%)	0	0	Hydrocortisone equivalent; dose ≥400 mg/day for ≥1 week; details about individual glucocorticoids and routes of administration not reported
Baker et al.^33^	Retrospective cohort	North America	245	7 days	67.3 (12.5)	146 (59.7%)	Not reported	245 (100%)	0	21 (8.6%)	Methyl‐prednisolone equivalent; 40 mg/day for 5 days; details about individual glucocorticoids and routes of administration not reported
Cole et al.^35^	Retrospective cohort	North America	54	12 months	70.5 (7.7)	1 (1.9%)	28.5 (4.6)	54 (100%)	0	24 (42.9%)	Prednisone equivalent; dose 85.5 ± 57 mg for 7.5 (5.5–10.25) days; details about individual glucocorticoids and routes of administration not reported
Johannesmeyer et al.^39^	Retrospective cohort	North America	209	8 months	77 (69–84)	112 (53.6%)	26.5 (22.6–31.2)	209 (100%)	0	49 (23.4%)	Prednisone equivalent; 128 mg/day, for a median (IQR) 4 (3–6) days; details about individual glucocorticoids and routes of administration not reported
George et al.^37^	Retrospective cohort	North America	190	3 months	66.1 (11.2)	98 (51.6%)	Not reported	190 (100%)	0	56 (29.5%)	Prednisone or equivalent; 40 mg for 5 days; details about individual glucocorticoids and routes of administration not reported
McGraw et al.^42^	Retrospective cohort	North America	1120	2 years 6 months	69 (12)	639 (57%)	29.4 (9.8)	1120 (100%)	0	349 (31.2%)	Prednisone equivalent; 86.0 mg/day; duration and details about individual glucocorticoids and routes of administration not reported
Burt et al.^34^	Prospective cohort	Australia	60	1 year 7 months	78.4 (11.9)	21 (35.3%)	28.1 (6.8)	47 (78%)	13 (22%)	7 (11.7%); 7 in exposed group and 0 in control group	Oral prednisolone for 78 days; 30 ± 6 mg/day for those with no diabetes and 26 ± 9 mg/day for those with diabetes at baseline
Delcampo et al.^36^	Prospective cohort	Australia	61	Not reported	71.2 (10.7)	37 (60.6%)	Not reported	61 (100%)	0	14 (23%)	Oral prednisolone 35.5 mg/day for 4.5 days
Habib et al.^38^	Prospective cohort	Asia	44	3 months	66.2 (8.2)	5 (11.3%)	31.4 (7.0)	23 (52%)	21 (48%)	44 (100%) (23 in exposed group and 21 in control group)	Intravenous hydrocortisone/oral prednisone; total dose 520 ± 302 mg/day for 18.8 ± 8.3 days
Roberts et al.^43^	Prospective cohort	Australia	136	4 days	78	Not reported	26.8	19 (14%)	117 (86%)	124 (91.2%); 7 in exposed and 117 in control group	Oral prednisolone≥20 mg or more daily for 4 days
Maltais et al.^19^	RCT	Multi‐region (Europe & Canada)	128	10 days	70.0 (8.4)	37 (19.9%)	Not reported	62 (48%)	66 (52%)	0	Oral prednisolone (30 mg every 12 h) for 72 h followed by prednisolone (40 mg daily) for 7 days
Sayiner et al.^16^	RCT	Multi‐region (Europe and Asia)	36	6 months	65.8 (1.8)	2 (5.5%)	Not reported	36 (100%)	0	0	IV methyl‐prednisolone; 0.5 mg/kg for 10 days
Leuppi et al.^40^	RCT	Europe	311	5 years	69.8 (11.0)	123 (39.6%)	Not reported	311 (100%)	0	0	IV methyl‐prednisolone or oral prednisone; 40 mg/day; either 5 or 14 days
Lichtblau et al.^41^	RCT	Asia	95	3 days	57 (8.0)	16 (16.8%)	25.4 (3.9)	52 (55%)	43 (45%)	1 (1.1%);0 in exposed and 1 in non‐exposed	Oral dexamethasone 8 mg/day for 3 days
Alia et al.^32^	RCT	Multi‐region (North & South America, Europe)	83	4 years	68.4 (10.2)	17 (20.5%)	Not reported	43 (52%)	40 (48%)	24 (28.9%); 15 in exposed and 9 in non‐ exposed	IV methylprednisolone 0.5 mg/kg every 6 h for 72 h, 0.5 mg/kg every 12 h on Days 4 through 6, 0.5 mg/kg daily for Day 7 through Day 10
Niewoehner et al.^20^	RCT	North America	271	6 months	67.7 (9.3)	3 (3.8%)	Not reported	160 (59%)	111 (41%)	28 (10.3%);23 in exposed and 5 in non‐ exposed	IV methylprednisolone (125 mg every 6 h for 72 h) followed by tapering schedule oral prednisone for 8 weeks or 12 days followed by 6 weeks placebo.
Abroug et al.^31^	RCT	Africa	217	10 days	69 (63–75)	26 (12.0%)	Not reported	111 (51%)	106 (49%)	30 (13.8%);17 in exposed and 13 in non‐ exposed	Oral prednisone (1 mg/kg daily) until discharge or maximum 10 days
Sivapalan et al.^14^	RCT	Europe	318	3 months	75 (68–82)	175 (55.1%)	23.9 (20.3–27.9)	318 (100%)	0	39 (12.3%)	IV methyl‐prednisolone; 80 mg/day for 14 days

Note: Prevalence was calculated for the exposed or intervention group only.

Abbreviations: BMI, body mass index; IQR, interquartile range; IV, intravenous; N/A, not available; RCT, randomised controlled trial; SD, standard deviation.

Of the selected 18 studies, eight were RCTs and 10 were observational studies. Among the eight RCTs, double blinding was used in six studies,^19^, ^20^, ^31^, ^32^, ^40^, ^41^ one was single‐blinded^16^ and one was an open‐label study.^14^ Among the 10 observational studies, four were prospective^34^, ^36^, ^38^, ^43^ and six were retrospective cohort studies.^13^, ^33^, ^35^, ^37^, ^39^, ^42^ There were no cross‐sectional or case–control studies. The included studies were published between 1999 and 2023 from 13 countries and 5 continents. Seven studies were based in North America, 1 in Africa, 2 in Asia, 3 in Australia, 2 in Europe, and 3 in multiple continents (Table 1).

Median (IQR) study sample size at baseline was 132 (61–245). Median age at baseline in all studies was 69 years and varied between 57 and 78 years, while the proportion of women ranged from 1.9% to 60.6% in studies where it was reported. The proportion of people with documented diagnosis of diabetes at baseline was between 0% and 100%, with a median of 13.1%. Four studies^13^, ^16^, ^19^, ^40^ reported that no people had diabetes at baseline while one study^38^ reported that all included people had diabetes at baseline. Details about the type or duration of diabetes at baseline were not reported in any of the studies. Four studies^14^, ^19^, ^20^, ^33^ reported the proportion of people using different types of maintenance therapy for COPD. The most used maintenance therapies were long‐acting ß2‐receptor agonists (43%–97% people) and long‐acting muscarinic receptor agonists (21–82% people) followed by inhaled corticosteroids (44%–60% people). Details on COPD exacerbations were reported in three studies. Niewoeher et al.^20^ reported that 64%–75% of the people had a hospitalisation for COPD exacerbation in the previous 2 years while George et al.^37^ reported that admission for COPD exacerbation in the previous 12 months was required among 43% people. Sivapalan et al.^14^ documented a mean rate of severe COPD exacerbation in the previous 12 months of 0.64 (95% CI 0.45–0.83).

The proportion of people exposed to systemic glucocorticoids varied between 14% and 100% (Table 1) and was 100% in 10 studies.^13^, ^14^, ^16^, ^33^, ^35^, ^36^, ^37^, ^39^, ^40^, ^42^ The duration of systemic glucocorticoid treatment was between 3 and 78 days. In one study^42^ treatment duration was not reported. The reported types of systemic glucocorticoids included prednisolone, prednisone, methylprednisolone, hydrocortisone and dexamethasone. Seven studies^13^, ^33^, ^35^, ^37^, ^39^, ^40^, ^42^ reported prednisone, hydrocortisone or methylprednisolone equivalents to standardise the dose but did not specify all glucocorticoids used. Doses ranged between 400 and 822 mg/day for hydrocortisone equivalents, 20–86 mg/day for prednisolone equivalents and 40–500 mg/day for methylprednisolone equivalents. The follow‐up duration ranged from 3 days to 5 years and was not reported in one study.^36^

Ascertainment of glycaemia and definitions of hyperglycaemia and diabetes are detailed in Tables 2 and 3, respectively. Different definitions of hyperglycaemia were used based on a specific cut‐off in glucose concentration or introduction or increase in the dose of glucose‐lowering therapy. Most studies defined hyperglycaemia as glucose concentration in venous blood or serum >10 mmol/L or > 11.1 mmol/L (Table 2). Two studies used fasting plasma glucose of ≥5.5 mmol/L or random plasma glucose of ≥7.8 mmol/L to define new hyperglycaemia^37^, ^40^ while one study used fasting plasma glucose ≥7.0 mmol/L.^14^ Three studies used an introduction or increase in the dose of glucose‐lowering therapy in conjunction with glucose concentration to define hyperglycaemia.^31^, ^32^, ^40^ Definition of hyperglycaemia was not provided in 4 studies.^16^, ^19^, ^20^, ^38^ Two studies^13^, ^14^ reported new‐onset diabetes and used established definitions in terms of blood glucose concentration or HbA1c (Table 3).

**TABLE 2:** Studies reporting new‐onset hyperglycaemia: Summary of outcome related information.

First author (year of publication)	*N* new cases of hyperglycaemia /*N* exposed	*N* new cases of hyperglycaemia /*N* non‐exposed	Definition of new‐onset hyperglycaemia
Upadhyay et al.^13^	42/64	N/A	Glucocorticoid‐induced hyperglycaemia defined as 2 or more readings of plasma glucose 140 mg/dL (or ≥7.8 mmol/L)
Baker et al.^33^	118/245	N/A	A blood glucose value of 180 mg/dL (10 mmol/L) to define an event based on the recommended target for hospitalized non‐critically ill people in the practice guidelines from the American Diabetes Association and the Endocrine Society.
Cole et al.^35^	17/54	N/A	Glucose readings greater than 180 mg/dL (10 mmol/L)
Johannesmeyer et al.^39^	97/209	N/A	Serum glucose concentration above 180 mg/dL (10 mmol/L)
George et al.^37^	96/190	N/A	Hyperglycaemia was defined as a fasting blood glucose 100 mg/dL (5.5 mmol/L) or random blood glucose of 140 mg/dL (7.8 mmol/L)
McGraw et al.^42^	446/1120	N/A	High maximum glucose was recorded as an indicator of whether the patient had any glucose concentration reading over 180 mg/dL (10 mmol/L) during hospitalization
Burt et al.^34^	28/47	1/13	Glucose of at least 200 mg/dL (11.1 mmol/L) during continuous glucose monitoring
Delcampo et al.^36^	10/61	N/A	Blood glucose concentration above 11.1 mmol/L
Habib et al.^38^	23/23	11/21	Reported but not defined
Roberts et al.^43^	15/19	33/117	Recorded blood glucose concentration above 10 mmol/L
Maltais et al.^19^	7/62	0/66	Reported but not defined
Sayiner et al.^16^	4/36	N/A	Reported but not defined
Leuppi et al.^40^	148/311	N/A	New or worsening hyperglycaemia was defined as fasting plasma glucose of 100 mg/dL (5.5 mmol/L) or greater, random plasma glucose of 140 mg/dL (7.8 mmol/L) or greater, an increase of 20% or more in daily doses of insulin, any increase in oral antidiabetic drugs, initiation of one or more antidiabetic therapeutic principles
Lichtblau et al.^41^	15/52	2/43	Blood glucose concentration above 11.1 mmol/L
Alia et al.^32^	20/43	10/40	Hyperglycaemia (the initiation of insulin therapy because of a blood glucose concentration > 120 mg/dL (6.7 mmol/L) in people without preexisting diabetes mellitus or increased doses of insulin in people with diabetes mellitus)
Niewoehner et al.^20^	24/160	4/111	Reported but not defined
Abroug et al.^31^	55/111	35/106	Blood glucose level ≥180 mg/dL (10 mmol/L) in people without pre‐existing diabetes or increase in initial insulin therapy
Sivapalan et al.^14^	24/318	N/A	Fasting plasma glucose ≥7.0 mmol/L

*Note*: American Diabetes Association guidelines: Umpierrez et al.^44^ American Diabetes Association.^45^

Abbreviation: N/A, not available.

**TABLE 3:** Studies reporting new‐onset diabetes: Summary of outcome related information.

First author (year of publication)	*N* new cases of diabetes in the exposed /*N* exposed	*N* new cases of diabetes in the non‐exposed/*N* non‐exposed	Prevalence of new‐onset diabetes	Definition of new‐onset diabetes
Upadhyay et al.^13^	5/64	N/A	7.8%	Diabetes defined as fasting glucose of >126 mg/dL or ≥7.00 mmol/L or HbA1c > 6.5% or a postprandial glycaemia ≥11.1 mmol/L or 200 mg/dL as per American Diabetes Association guidelines.
Sivapalan et al.^14^	12/318	N/A	4.3%	New onset diabetes defined as HbA1c ≥48 mmol/mol.

Abbreviation: N/A, not applicable.

### 3.3 Prevalence of GIH

**FIGURE 2:**
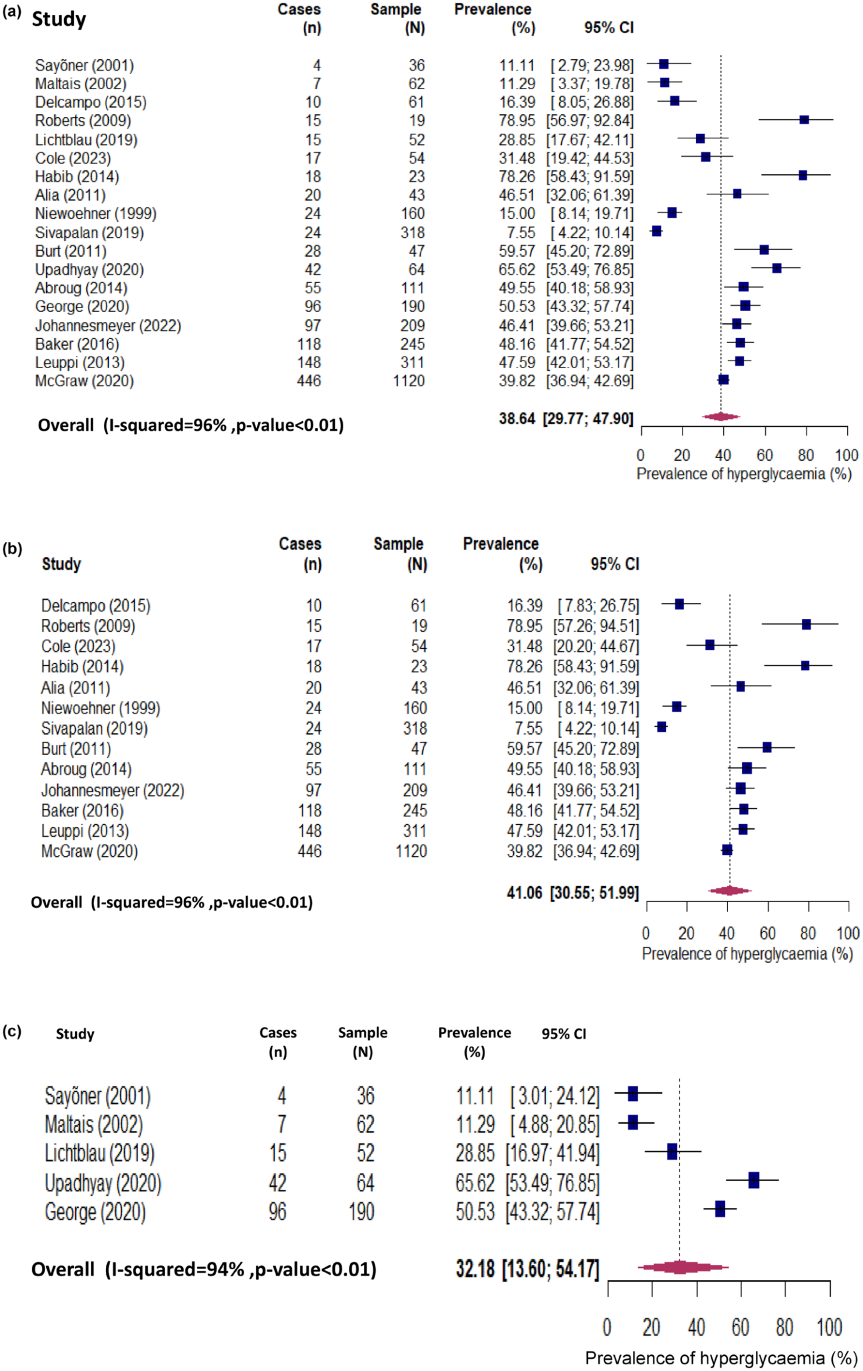
(a) Prevalence of glucocorticoid‐induced hyperglycaemia in all 18 studies. (b) Prevalence of glucocorticoid‐induced hyperglycaemia in 13 studies which included people with and those without diabetes at baseline. (c) Prevalence of glucocorticoid‐induced hyperglycaemia in 5 studies, which included only people without diabetes at baseline.

Eighteen studies reported data allowing us to calculate prevalence estimates for GIH in the exposed people. The pooled prevalence was 38.6% (95% CI 29.8%–47.9%), with a significant heterogeneity between the studies, *I* ^2^ = 96%, *p* < 0.010 (Figure 2). In the 13 studies which included people with or without diabetes at baseline (i.e. the same study included people who had diabetes and people who did not have diabetes), the prevalence was 41.1% (95% CI 30.6%–52.0%) (Figure 2b), while in the five studies which included only people without diabetes at baseline the prevalence was 32.2% (95% CI 13.6%–54.2%) (Figure 2c).

### 3.4 Prevalence of GID: narrative synthesis

Relevant data were reported in two studies including 382 exposed people of whom 17 developed new‐onset diabetes (Table 3). Calculated prevalence of new‐onset diabetes was 4.3% in Sivapalan et al.^14^ and 7.8% in Upadhyay et al.^13^ (Table 3).

### 3.5 Subgroup analysis

**FIGURE 3:**
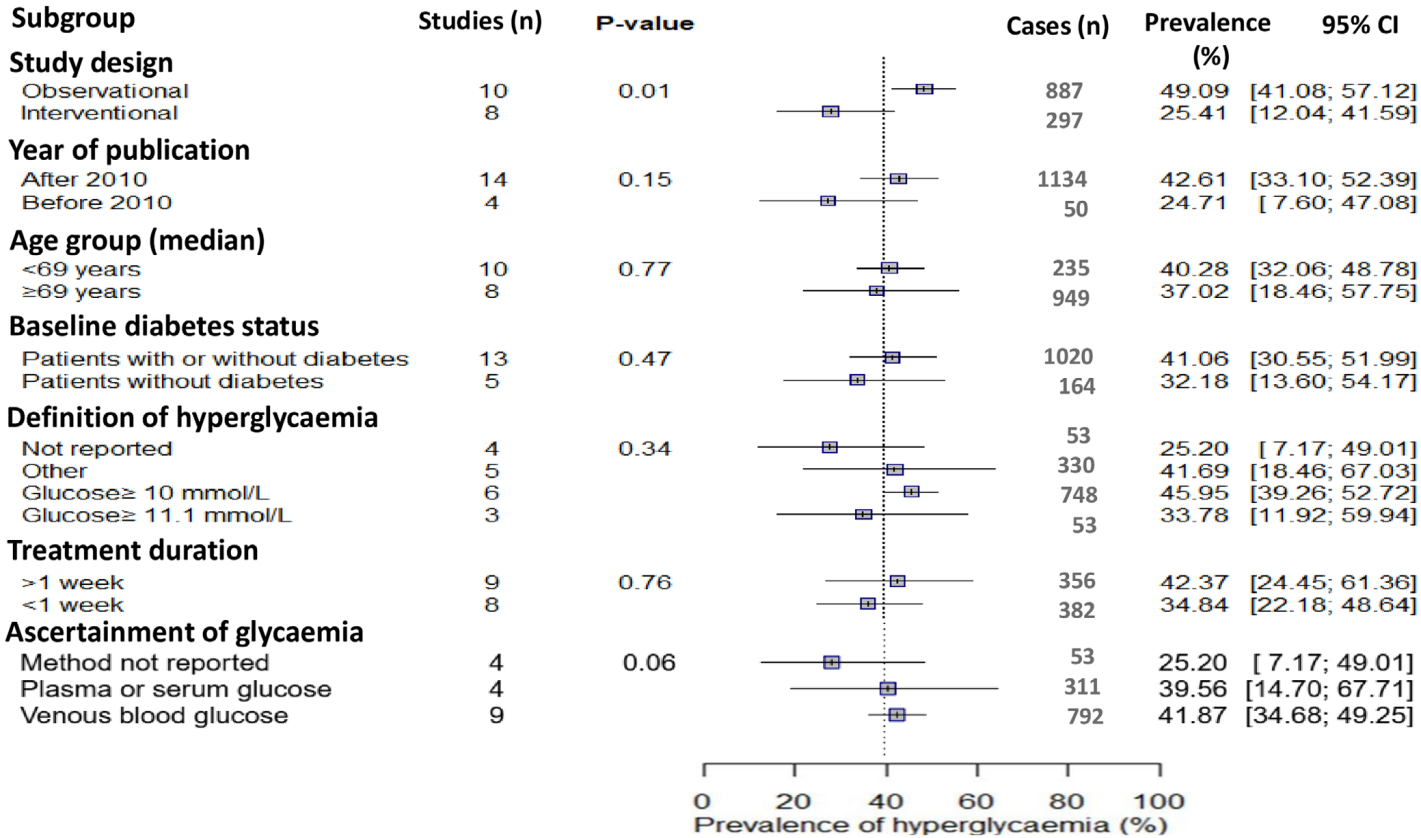
Prevalence of glucocorticoid‐induced hyperglycaemia by subgroup. The *p*‐values are tests of significance for difference between the subgroup effects. In the definition of hyperglycaemia, category other included definitions based on fasting plasma glucose ≥5.5 mmol/L or ≥7.0 mmol/L or random plasma glucose ≥7.8 mmol/L. In the analysis of treatment duration, one study (McGraw et al.^42^), one study was excluded, hence 17 rather than 18 studies. This was done because no details on treatment duration were reported in this study. In the analysis of the ascertainment of glycaemia, one study was excluded (Burt et al.^34^), hence 17 rather than 18 studies. This was done because this study used continuous glucose monitoring, a method not belonging to any of the categories. The analysis could be done with three or more studies per category.

We conducted a pre‐planned subgroup analysis for the 18 studies focussing on the prevalence of GIH. Most of the variation between the studies was attributable to true heterogeneity rather than chance as indicated by *I* ^2^ = 96%, *p* < 0.010. The heterogeneity was partly explained by differences in study design (Figure 3) with significantly higher prevalence of GIH in observational vs interventional studies, 49.1% (95% CI 41.1%–57.1%) versus 25.4% (95% CI 12.0%–41.6%), *p* < 0.010 (Figure 3). Other study‐level characteristics including age, baseline diabetes status, publication year, treatment duration, definition of hyperglycaemia and method to ascertain glycaemia did not substantially contribute to the observed heterogeneity (Figure 3).

### 3.6 Relative risk of new‐onset hyperglycaemia

**FIGURE 4:**
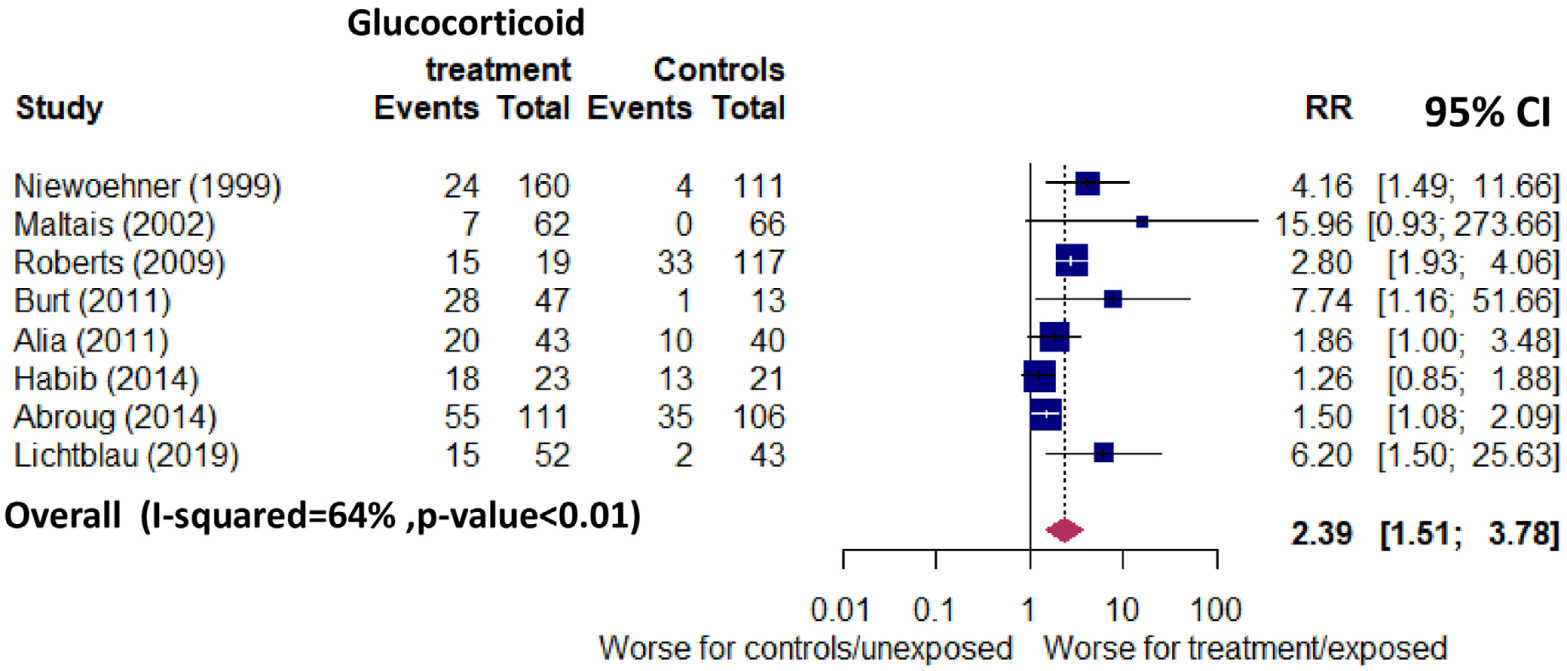
Relative risk (95% CI) of new‐onset hyperglycaemia in people exposed to systemic glucocorticoids versus non‐exposed in 8 studies which reported data to calculate relative risk.

We calculated RR of new‐onset hyperglycaemia based on reported data on the number of new cases in the exposed and unexposed groups and the total number of people exposed and unexposed to systemic glucocorticoids. Relevant data were reported in eight studies including 1034 people of whom 283 developed new‐onset hyperglycaemia. The pooled RR was 2.39 (95% CI 1.51–3.78) with a moderate heterogeneity, *I* ^2^ = 64%, *p* < 0.01 (Figure 4). We conducted a sensitivity analysis excluding one study which included only people without diabetes at baseline and the pooled RR remained unchanged (Figure S1).

### 3.7 Publication bias

To evaluate publication bias in the 18 studies focussing on the prevalence of GIH, we created a funnel plot whereby calculated prevalence (i.e. proportion) for each study was plotted against its standard error. There was funnel plot asymmetry suggesting that smaller studies were more likely to yield more extreme results (Figure S2). However, Egger's test for publication bias was not statistically significant, *p* = 0.84. We also evaluated publication bias in the 8 studies which reported relevant data to calculate the RR for new onset GIH. The funnel plot also showed asymmetry (Figure S3) but Egger's test was not statistically significant (*p* = 0.061).

### 3.8 Risk of bias

Our risk of bias assessment identified some concerns in the domain pertaining to the bias in selection of the reported results in seven of eight included RCTs (Figure S4). Overall, in one RCT, some concerns about bias were identified due to bias in the selection of the reported results while seven were deemed to have no risk of bias. None of the RCTs was considered to have high risk of bias. Among the 10 observational studies, seven had a score of 7 (out of 8) and three had a score of 8 (Table S4). We conducted a sensitivity analysis excluding studies in which some concerns about the risk of bias were identified in any of the domains and pooled prevalence of GIH was 45.4% (95% CI 34.3%–56.7%) (Figure S5), which is somewhat higher than the pooled prevalence across all 18 studies 38.6% (95% CI 29.8%–47.9%) (Figure 2a).

## 4 DISCUSSION

This systematic review provides further support for a high prevalence of GIH in people with COPD treated with systemic glucocorticoids and significantly elevated risk of new‐onset hyperglycaemia in people with COPD receiving systemic glucocorticoids vs those not receiving systemic glucocorticoids. The prevalence of GIH was 38.6% while being treated with systemic glucocorticoids for COPD was associated with 2.4 times higher risk of new‐onset hyperglycaemia in people exposed versus non‐exposed to glucocorticoid treatment. Prevalence of GID was 4.3%^14^ and 7.8%^13^ in two studies with available data. We demonstrated that high heterogeneity of the estimates of prevalence of GIH between the studies was partly explained by differences in study design with higher prevalence in observational vs interventional studies, 49.1% (95% CI 41.1%–57.1%) versus 25.4% (95% CI 12.0%–41.6%), *p* < 0.010 while other study characteristics including age, diabetes status at baseline, treatment duration, method of ascertainment of glycaemia, definition of hyperglycaemia and year of publication did not contribute significantly to the observed heterogeneity. We found evidence of publication bias for both the prevalence of GIH and RR of new‐onset hyperglycaemia in people treated with systemic glucocorticoids vs those not treated. However, the model with RR may have been underpowered as it contained only 8 studies. Thus, the non‐significant Egger's test with a funnel plot asymmetry may indicate insufficient statistical power to detect publication bias.

Kulkarni et al.^46^ conducted a systematic review and meta‐analysis of adverse events associated with systemic glucocorticoids and reported a pooled prevalence of incidental hyperglycaemia of 22% (95% CI 9%–35%) in nine RCTs including trial arms of people who received glucocorticoids for COPD or community acquired pneumonia. The risk of hyperglycaemia associated with glucocorticoid treatment was not specifically reported for people with COPD, but the authors reported an odds ratio (OR) of 2.13 (95% CI 1.66–2.72) versus placebo for hyperglycaemia in people treated with systemic glucocorticoids for a variety of conditions in 15 double‐blinded placebo‐controlled RCTs.^46^ A meta‐analysis of 13 observational studies (predominantly retrospective and cross‐sectional) by Liu et al.^6^ showed that 32.6% of the people without diabetes develop hyperglycaemia following treatment with systemic glucocorticoids. However, this study analysed all indications together and respiratory disease as a composite end‐point rather than COPD was an indication for glucocorticoids in one of the studies.^6^ In a meta‐analysis of 8 RCTs including people with COPD and community acquired pneumonia,^7^ Breaky et al. compared the risk of hyperglycaemia in people randomised to systemic glucocorticoid treatment vs placebo and reported a relative risk of 1.72 (95% CI 1.50–2.04). A Cochrane systematic review focusing on systemic glucocorticoids for acute exacerbations of COPD^47^ reported RR = 4.95 (95% CI 2.47,9.91) vs placebo in 4 RCTs. However, there was no information about baseline diabetes status in some of these studies and one study used glycosuria to assess glycaemic adverse events as a proxy for blood glucose.^47^ The observed discrepancies between our findings and those reported in previous meta‐analyses are likely explained by the differences in inclusion criteria with respect to indication for glucocorticoids, study design, and documentation of diabetes status at baseline.

Unlike some previous meta‐analyses which were limited to RCTs,^7^, ^47^ we included RCTs and observational studies. Thus, it is possible that the inherent methodological limitations in observational studies such as chance, bias and confounding have affected our findings. Differences in characteristics of the people who did and those who did not receive systemic glucocorticoid therapy in observational studies may have introduced bias in our results. People with severe symptoms were more likely to receive systemic glucocorticoids than those with mild or moderate symptoms. Propensity score matching (PSM) is a statistical technique used in pharmacoepidemiology which mimics randomisation.^48^ PSM aims to balance the treatment groups according to baseline characteristics which are considered to be associated with the outcome while a propensity score represents a conditional probability of receiving treatment based on given characteristics.^48^ None of the included observational studies used PSM thus suggesting a possibility of bias. We assessed the prevalence of GIH by study design and observed a higher prevalence in observational vs interventional studies, 49.1% (95% CI 41.1%–57.1%) versus 25.4% (95% CI 12.0%–41.6%), respectively, *p* < 0.010. This difference between the subgroups was statistically significant suggesting that the observed heterogeneity between all included studies may in part be explained by the differences in study design.

We assessed the risk of bias using standardised and validated tools, namely NOS^26^ for observational studies and RoB2^25^ for RCTs. We did not identify any RCTs with a high risk of bias and observational studies scored 7 or 8 out of 8. Among RCTs, some concerns about bias were identified in 6 of 8 studies in the domain related to the selection of the reported results. We performed a sensitivity analysis of the pooled prevalence of GIH excluding studies with some concerns about bias and observed a higher prevalence compared to all 18 included studies, 45.4% (95% CI 34.3%–56.7%) versus 38.6% (95% CI 29.8%–47.9%). This difference is likely to reflect the previously discussed differences in study design because the excluded studies were RCTs. The remaining 12 studies in this sensitivity analysis consisted of 10 observational studies and 2 RCTs, which may in part account for the pooled estimate being in magnitude closer to that of observational studies.

Furthermore, the RoB2 tool is designed to assess bias with respect to the primary outcome which tended to be a COPD‐related end point rather than hyperglycaemia or other safety outcomes. Hence, bias assessment in domains related to measuring the outcome and missing outcome data might have been underestimated. Indeed, we observed that 4 studies did not report the method of ascertaining glycaemia or the definition of hyperglycaemia which might have introduced bias but would not have been captured using RoB2 tool as it focuses on the primary outcome. This error is likely to have been non‐differential because both exposed and non‐exposed people had glucose measured using the same method. The direction of error remains unknown in the absence of definition of hyperglycaemia; that is, the prevalence of GIH in these studies could have been underestimated or overestimated.

Methods of ascertainment of glycaemia and definitions of hyperglycaemia varied across the studies. We observed the highest prevalence of GIH in studies, which used venous blood glucose to assess glycaemia and in studies where hyperglycaemia was defined as blood glucose concentration ≥10 mmol/L. However, the differences between the subgroups of ascertainment of glycaemia and definitions of hyperglycaemia were not statistically significant and did not explain the heterogeneity in the prevalence between all 18 studies.

Immortal time bias is a systematic error which occurs in pharmacoepidemiologic studies in the exposed participants when the period before the start of exposure to medication is misclassified as exposed time.^49^ This leads to biased estimates but can be addressed by using time‐varying exposure which allows for a person to be considered non‐exposed until the first prescription after which the same person is considered exposed.^49^ The included studies did not use time to event analysis. Therefore, we could not assess the effect of the immortal time bias on our results.

Detection bias in this systematic review cannot be excluded. It is possible that the people who received systemic glucocorticoids were more likely to seek medical attention compared to their counterparts who did not receive systemic glucocorticoids. Therefore, people who received systemic glucocorticoids were more likely to have blood glucose concentration measured and hyperglycaemia documented. It is also possible that people who did not receive systemic glucocorticoids sought medical attention more frequently for the underlying COPD than those who received systemic glucocorticoids, but this is less likely to have occurred because the severity of COPD was comparable in exposed and non‐exposed people in RCTs while in 7 of 10 observational studies, all people were exposed.

It is possible that new‐onset hyperglycaemia and diabetes were associated with a pre‐existing metabolic dysfunction including insulin resistance, reduced utilisation of glucose by the peripheral tissues, increased hepatic glucose output and beta‐cell dysfunction related to previous courses of systemic glucocorticoids.^50^ However, we could not assess this based on the reported data.

Several analyses considered in our protocol were not conducted because of the lack of relevant data. Thirteen of the 18 selected studies included people with and those without diabetes at baseline and the numbers of people with new‐onset hyperglycaemia were not reported by diabetes status but for all exposed and all non‐exposed people. Five studies included only people without diabetes. Therefore, we could not calculate the prevalence of GIH by diabetes status but did so for people with or without diabetes versus people without diabetes. As expected, we observed a higher prevalence of GIH in the studies which included people with and those without diabetes.

New‐onset hyperglycaemia was reported only in the exposed people in 10 of 18 studies while eight studies reported new‐onset hyperglycaemia for both exposed and the unexposed people allowing us to calculate RR of new‐onset hyperglycaemia associated with glucocorticoids vs no glucocorticoid treatment in these 8 studies. We were not able to stratify the analysis of RR of new‐onset hyperglycaemia associated with glucocorticoids by baseline diabetes status because among these eight studies, only one was conducted in people without diabetes at baseline (i.e. did not include people with diabetes at baseline). Of the remaining seven studies, every study included people with and those without diabetes at baseline and the numbers of people with new‐onset hyperglycaemia were not reported by diabetes status. Among the studies which included people with and people without diabetes at baseline, no details of baseline glucose control were reported and the definition of hyperglycaemia did not differ according to diabetes status. Therefore, it was not possible to quantify the extent to which hyperglycaemia was driven by glucocorticoids vs suboptimal glucose control related to pre‐existing diabetes.

Since new‐onset diabetes was reported in only two studies, we provided a narrative synthesis of calculated prevalence of GID. The cases of new‐onset diabetes were not reported in non‐exposed groups in these two studies. Therefore, we could not calculate RR for new‐onset diabetes.

We planned to assess the incidence rate of GIH and GID and model the dose–response relationship between systemic glucocorticoid use and new‐onset diabetes and hyperglycaemia. However, none of the included studies provided data required for these analyses. We were not able to model the dose–response relationship because of the lack of explicit details on the dose, frequency and duration of glucocorticoid treatment required for this modelling. Specifically, such an analysis would require incidence of GIH or diabetes to be reported for three different exposure levels in each study. Incidence rates were not reported in any of the studies and there was one exposure level in the included studies.

We planned to analyse if the pooled estimates varied by maintenance treatment for COPD or by BMI but did not perform these analyses as this information was not available in most studies; information on maintenance treatment was available in only four studies while BMI was reported in eight studies. Since a systematic review of the risk of GIH and GID associated with inhaled corticosteroids for COPD^51^ has been recently published, we have not included inhaled corticosteroids in this review.

In conclusion, our findings suggest that in people with COPD, the prevalence of GIH is high, and that systemic glucocorticoid therapy markedly increases the risk of hyperglycaemia. It is possible that our findings are affected by publication bias. Clinicians providing care for people with COPD who are treated with systemic glucocorticoids should consider measuring blood glucose before starting systemic glucocorticoids and throughout the treatment course to detect and manage GIH.

## FUNDING INFORMATION

None.

## CONFLICT OF INTEREST STATEMENT

All authors declare no conflict of interest.

## Supporting information


**Data S1:** Supporting Information.

## Data Availability

The data that support the findings of this study are available from the corresponding author upon reasonable request.
