# Supplementary material for: Glucocorticoid treatment and new‐onset hyperglycaemia and diabetes in people living with chronic obstructive pulmonary disease: A systematic review and meta‐analysis
Source: Diabet Med. 2024 Dec 6;42(3):e15475. doi: 10.1111/dme.15475 (PMC11823367; doi:10.1111/dme.15475)
Supplement: Supplementary file 1 — Data S1: Supporting Information. [file DME-42-e15475-s001.docx]

**List of supplementary tables**

Table S1 Systematic review search strategies

Table S2 Glucocorticoids and their prednisolone equivalent dose

Table S3 List of all variables considered for extraction

Table S4 Quality assessment of observational studies using Newcastle-Ottawa scale

**List of supplementary figures**

Figure S1 Relative risk for new-onset hyperglycaemia in people exposed to systemic glucocorticoids *vs* non-exposed excluding one study where only patients without diabetes at baseline were included (sensitivity analysis)

Figure S2 Publication bias: prevalence of glucocorticoid-induced hyperglycaemia

Figure S3 Publication bias: relative risk of new-onset hyperglycaemia in people exposed to systemic glucocorticoids *vs* non-exposed

Figure S4 Risk of bias assessment in randomised controlled trials using Cochrane Risk of Bias RoB2 tool

Figure S5 Prevalence of glucocorticoid-induced hyperglycaemia excluding studies identified as “some concerns” in the risk of bias assessment (sensitivity analysis)

**Other supplementary material**

PRISMA checklist

**Table S1 Systematic review search strategies**

| PubMed | ("steroid"[Title/Abstract] OR "Steroids" [MeSH:NoExp] OR "corticosteroid"[Title/Abstract] OR "glucocorticoid"[Title/Abstract] OR "prednisolone"[Title/Abstract] OR "prednisone"[Title/Abstract] OR "methylprednisolone"[Title/Abstract] OR "hydrocortisone"[Title/Abstract] OR "dexamethasone"[Title/Abstract] OR "budesonide"[Title/Abstract] OR "cortisone"[Title/Abstract] OR "deflazacort"[Title/Abstract] OR "plenadren"[Title/Abstract] OR "triamcinolone"[Title/Abstract]) AND "humans"[MeSH Terms]  AND  (("diabetes"[Title/Abstract] OR "diabetes mellitus"[Title/Abstract] OR "hyperglycemia"[Title/Abstract] OR "Hyperglycemia" [MeSH:NoExp] OR "hyperglycaemia"[Title/Abstract] OR "steroid induced diabetes"[Title/Abstract] OR "steroid induced hyperglycemia"[Title/Abstract] OR "corticosteroid induced diabetes"[Title/Abstract] OR "corticosteroid induced hyperglycemia"[Title/Abstract] OR ("glycemic control" [Title/Abstract]) OR "glucocorticoid induced diabetes"[Title/Abstract] OR "glucocorticoid induced hyperglycemia"[Title/Abstract])  AND "humans"[MeSH Terms]) |
| --- | --- |

| EMBASE | 1 steroid.ab,ti,tw.  2 corticosteroid.ab,ti,tw.  3 glucocorticoid.ab,ti,tw.  4 prednisolone.ab,ti,tw.  5 prednisone.ab,ti,tw.  6 methylprednisolone.ab,ti,tw.  7 hydrocortisone.ab,ti,tw.  8 dexamethasone.ab,ti,tw.  9 budesonide.ab,ti,tw.  10 cortisone.ab,ti,tw.  11 deflazacort.ab,ti,tw.  12 plenadren.ab,ti,tw.  13 triamcinolone.ab,ti,tw.  14 1 or 2 or 3 or 4 or 5 or 6 or 7 or 8 or 9 or 10 or 11 or 12 or 1 3  15 hyperglycemia.ab,ti,tw.  16 hyperglycaemia.ab,ti,tw.  17 glycemic control.ab,ti,tw.  18 diabetes.ab,ti,tw.  19 diabetes mellitus.ab,ti,tw.  20 steroid induced diabetes.ab,ti,tw.  21 steroid induced hyperglycemia.ab,ti,tw.  22 corticosteroid induced diabetes.ab,ti,tw.  23 corticosteroid induced hyperglycemia.ab,ti,tw.  24 glucocorticoid induced diabetes.ab,ti,tw.  25 glucocorticoid induced hyperglycemia.ab,ti,tw.  26 15 or 16 or 17 or 18 or 19 or 20 or 21 or 22 or 23 or 24 or 2  27 14 and 26  28 limit 27 to (human and (adult <18 to 64 years> or aged <65+ years>)) |
| --- | --- |

| Cochrane Library | #1 (steroid OR corticosteroid OR glucocorticoid):ti,ab,kw  #2 (prednisolone):ti,ab,kw  #3 (prednisone):ti,ab,kw  #4 (methylprednisolone):ti,ab,kw  #5 (hydrocortisone):ti,ab,kw  #6 (dexamethasone):ti,ab,kw  #7 (budesonide):ti,ab,kw  #8 (cortisone):ti,ab,kw  #9 (deflazacort):ti,ab,kw  #10 (plenadren):ti,ab,kw  #11 (triamcinolone):ti,ab,kw  #12 #1 OR #2 OR #3 OR #4 OR #5 OR #6 OR #7 OR #8 OR #9 OR #10 OR #11  #13 MeSH descriptor: [Glucocorticoids] explode all trees  #14 (hyperglycemia):ti,ab,kw  #15 (hyperglycaemia):ti,ab,kw  #16 (glycemic control):ti,ab,kw  #17 (diabetes):ti,ab,kw  #18 (diabetes mellitus):ti,ab,kw  #19 (steroid induced diabetes):ti,ab,kw  #20 (steroid induced hyperglycemia):ti,ab,kw  #21 (corticosteroid induced diabetes):ti,ab,kw  #22 (corticosteroid induced hyperglycemia):ti,ab,kw  #23 (glucocorticoid induced diabetes):ti,ab,kw  #24 (glucocorticoid induced hyperglycemia):ti,ab,kw  #25 MeSH descriptor: [Hyperglycemia] this term only  #26 #13 OR #14 OR #15 OR #16 OR #17 OR #18 OR #19 OR #20 OR #21 OR #22 OR #23 OR #24 OR #25  #27 #12 AND #26  #28 #27 in Trials |
| --- | --- |

| ClinicalTrials.gov | steroid OR corticosteroid OR glucocorticoid OR prednisolone OR hydrocortisone OR dexamethasone OR budesonide OR cortisone OR deflazacort OR plenadren OR triamcinolone \| Hyperglycemia \| Adult, Older Adult |
| --- | --- |

**Table S1 Glucocorticoids and their prednisolone equivalent dose**

| **Name** | **Dose (mg)** | **Systemic routes of administration to be included in the analysis** |
| --- | --- | --- |
| Budesonide | 0.55 | PO |
| Cortisone | 25 | PO, IV, IM |
| Deflazacort | 6 | PO |
| Dexamethasone | 0.8 | PO, IV, IM |
| Hydrocortisone | 20 | PO, IV, IM |
| Methylprednisolone | 4 | PO, IV |
| Plenadren | 20 | PO, IV |
| Prednisolone | 5 | PO |
| Prednisone | 5 | PO |
| Triamcinolone | 4 | IM |

The conversions are based on the British National Formulary (National Institute for Health and Care Excellence. British National Formulary. London: NICE, 2022.).

**Table S3 List of all variables considered for data extraction**

Study details and population

| **Variable name** | **Variable type/description** | **List of categories for categorical variables** |
| --- | --- | --- |
| First author’s name | Text | N/A |
| Year of publication | Integer number; continuous | N/A |
| Study name | Text | N/A |
| Baseline study year | Integer number; continuous | N/A |
| Country/countries | Text; free text | N/A |
| Continent/region | Text; categorical | Europe; North America; South America; Asia; Africa; Australia; multiregional |
| Study setting | Text; categorical | inpatient; outpatient primary care; outpatient secondary care; other; not reported |
| Is it clear which study design was used? | Text; categorical | yes; no |
| Study design | Text; categorical | randomised controlled trial; prospective cohort study; retrospective cohort study; case-control study; non-randomised trial of intervention; cross-sectional; unknown |
| If this is a trial, has it been randomised? | Text; categorical | yes; no |
| Blinding | Text; categorical | patient; assessor; patient and assessor; unknown; not applicable |
| Total N at baseline | Integer number; continuous | N/A |
| N (%) exposed to glucocorticoids | Integer number (%); continuous | N/A |
| N (%) non-exposed to glucocorticoids | Integer number (%); continuous | N/A |
| Unit of time of follow-up duration | Text; categorical | days; weeks; months; years |
| Follow-up duration (unit of time), mean | Number with a decimal place; continuous | N/A |
| Follow-up duration (unit of time), SD | Number with a decimal place; continuous | N/A |
| Follow-up duration (unit of time), median | Number with a decimal place; continuous | N/A |
| Follow-up duration (unit of time), IQR | Number; range | N/A |
| Age (years), mean | Number with a decimal place; continuous | N/A |
| Age (years), SD | Number with a decimal place; continuous | N/A |
| Age (years), median | Number with a decimal place; continuous | N/A |
| Age (years), IQR | Number; range | N/A |
| **Variable name** | **Variable type/description** | **List of categories for categorical variables** |
| Sex, N (%) female | Integer number (%); continuous | N/A |
| BMI (kg/m^2^), mean | Number with a decimal place; continuous | N/A |
| BMI (kg/m^2^), SD | Number with a decimal place; continuous | N/A |
| BMI (kg/m^2^), median | Number with a decimal place; continuous | N/A |
| BMI (kg/m^2^), IQR | Number; range | N/A |
| Reported frequency of COPD exacerbations | Free text | N/A |
| Have patients used maintenance therapy for COPD? | Text; categorical | yes; no; not reported |
| In patients using maintenance therapy for COPD, what was it? | Text; categorical | beta 2 agonists; muscarinic agonists; inhaled corticosteroids alone; inhaled corticosteroids combined with beta 2 agonists or muscarinic agonists; other |
| If other maintenance therapy for COPD was used, what was it? | Free text | N/A |
| N (%) patients with diabetes at baseline | Integer number (%); continuous | N/A |
| Duration of diabetes (years) if diabetes status at baseline is positive, mean | Number with a decimal place; continuous | N/A |
| Duration of diabetes (years) if diabetes status at baseline is positive, SD | Number with a decimal place; continuous | N/A |
| Duration of diabetes (years) if diabetes status at baseline is positive, median | Number with a decimal place; continuous | N/A |
| Duration of diabetes (years) if diabetes status at baseline is positive, IQR | Number; range | N/A |
| Diabetes type at baseline | Text; categorical | type 1; type 2; type 1 and type 2; unknown |

Exposure

| **Variable name** | **Variable type** | **List of categories for categorical variables** |
| --- | --- | --- |
| Name of glucocorticoid | Free text | N/A |
| Was the dose reported in any other way than the exact mg/day? | Text; categorical | yes; no |
| If the dose was not reported as mg/day, how was it reported (min to max, greater than x, any other way)? | Free text | N/A |
| Number of exposure categories | Integer number; categorical | 1; 2; 3; 4; 5 |
| Reference categories contains only non-exposed to glucocorticoids | Text; categorical | yes; no |
| Tapering regimen reported | Text; categorical | yes; no |
| N participants for every exposure category | Integer number, continuous | N/A |
| Dose of glucocorticoid (mg) for every exposure category | Integer number, continuous | N/A |
| Frequency of administration in 24h for every exposure category | Integer number; categorical | 1; 2; 3; 4; continuous; not reported |
| Route of administration for every exposure category | Text; categorical | oral; intravenous; intramuscular; oral and intravenous; oral and intramuscular; intravenous and intramuscular; oral and intravenous and intramuscular; concurrent systemic and topical; systemic but specific type not reported |
| Tapering regimen as reported for every exposure category | Free text | N/A |
| Unit of exposure duration | Text; categorical | days; weeks; months; years |
| Glucocorticoid treatment duration in unit of time (mean) | Number with a decimal place; continuous | N/A |
| Glucocorticoid treatment duration in unit of time (SD) | Number with a decimal place; continuous | N/A |
| Glucocorticoid treatment duration in unit of time (median) | Number with a decimal place; continuous | N/A |
| Glucocorticoid treatment duration in unit of time (IQR) | Number; range | N/A |

Outcome

| **Variable name** | **Variable type** | **List of categories for categorical variables** |
| --- | --- | --- |
| For studies reporting prevalence: reported N (%) people with glucocorticoid-induced diabetes | Integer number (%), continuous | N/A |
| For studies reporting prevalence of glucocorticoid-induced diabetes: what is the denominator? | Text; categorical | all people exposed to glucocorticoids in the study; other; not reported |
| For studies reporting prevalence of glucocorticoid-induced diabetes: if other selected as denominator, what was it? | Free text | N/A |
| For studies reporting prevalence: reported N (%) people with glucocorticoid-induced hyperglycaemia | Integer number (%), continuous | N/A |
| For studies reporting prevalence of glucocorticoid-induced hyperglycaemia: what is the denominator? | Text; categorical | all people exposed to glucocorticoids in the study; other; not reported |
| For studies reporting prevalence of glucocorticoid-induced hyperglycaemia: if other selected as denominator, what was it? | Free text | N/A |
| New diabetes (glucocorticoid-induced diabetes) in patients without diabetes at baseline reported: yes/no | Text; categorical | yes; no |
| N (%) patients with new diabetes among non-exposed to glucocorticoids | Integer number (%), continuous | N/A |
| Reported N (%) of new diabetes cases for each exposure category | Integer number (%), continuous | N/A |
| Reported RR/HR/OR and 95% CI for new diabetes compared to non-exposed to glucocorticoids for each exposure category | Number with decimal places (range) | N/A |
| New hyperglycaemia (glucocorticoid-induced hyperglycaemia) reported: yes/no | Text; categorical | yes; no |
| N (%) patients with new hyperglycaemia among non-exposed to glucocorticoids | Integer number (%), continuous | N/A |
| N (%) of new hyperglycaemia for each exposure category | Integer number (%), continuous | N/A |
| RR/HR/OR and 95% CI for new hyperglycaemia compared to non-exposed to glucocorticoids for each exposure category | Number with decimal places (range) | N/A |
| New glucose-lowering treatment in response to new diabetes or hyperglycaemia | Text; categorical | yes; no; unknown |
| Name of new glucose-lowering treatment in response to new diabetes or hyperglycaemia | Free text | N/A |
| Category of new glucose lowering treatment | Text; categorical | sulphonylurea; insulin; sulphonylurea and insulin; other; none; unknown |
| Definition of new diabetes as reported in the article | Free text | N/A |
| Definition of new hyperglycaemia as reported in the article | Free text | N/A |
| Ascertainment of new diabetes | Text; categorical | point of care capillary blood glucose;  laboratory HbA1c;  laboratory fasting plasma glucose;  laboratory venous blood glucose;  oral glucose tolerance test;  self-report;  health records;  interstitial glucose from a flash or continuous glucose monitor;  other;  unknown;  multiple assessment methods |
| Ascertainment of new hyperglycaemia | Text; categorical | point of care capillary blood glucose;  laboratory HbA1c;  laboratory fasting plasma glucose;  laboratory venous blood glucose;  oral glucose tolerance test;  self-report;  health records;  interstitial glucose from a flash or continuous glucose monitor;  other;  unknown;  multiple assessment methods |
| If multiple methods, which ones? | Free text | N/A |

Abbreviations: N/A- not applicable; BMI- body mass index; HbA1c- glycated haemoglobin A1c; SD- standard deviation; IQR- interquartile range; RR- risk ratio; HR- hazard ratio; OR- odds ratio; CI- confidence interval

**Table S4 Quality assessment of observational studies using Newcastle-Ottawa scale**

| **Author, year of publication** | **Selection** | | | **Comparability** | **Outcome** | | **Total**  **(8*****)** |
| --- | --- | --- | --- | --- | --- | --- | --- |
|  | Representativeness of exposed cohort (*) | Selection of non-exposed cohort (*) | Ascertainment of exposure (*) | (**) | Assessment of outcome (*) | Adequacy of follow up (**) |  |
| Burt,2011 | * | * | * | * - | * | * * | ******* (7) |
| Habib, 2014 | * | * | * | * - | * | * * | ******* (7) |
| Upadhyay, 2020 | * | * | * | * - | * | * * | ******* (7) |
| McGraw, 2020 | * | * | * | * - | * | * * | ******* (7) |
| George, 2020 | * | * | * | * - | * | * * | ******* (7) |
| Baker, 2016 | * | * | * | * - | * | * * | ******* (7) |
| Roberts, 2009 | * | - | * | * * | * | * * | ******* (7) |
| Cole, 2023 | * | * | * | * * | * | * * | ******** (8) |
| Johannesmeyer, 2022 2022 | * | * | * | * * | * | * * | ******** (8) |
| Del Campo, 2015 | * | * | * | * * | * | * * | ******** (8) |

Comparability assessed as the following: Demonstration that outcome of interest was not present at start of study and comparability of cohorts on the basis of the design or analysis. Adequacy of follow up assessed as the following: Was follow-up long enough for outcomes to occur and adequacy of follow up of cohorts?


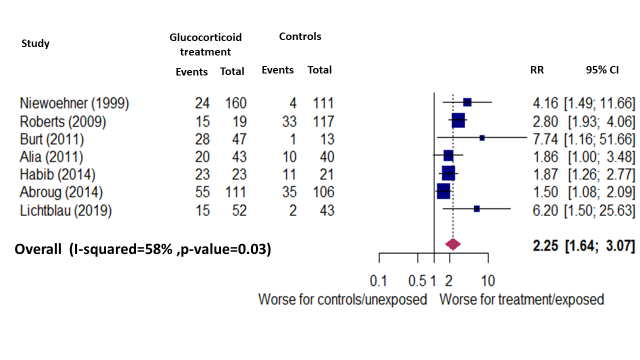


**Figure S1 Relative risk for new-onset hyperglycaemia in people exposed to systemic glucocorticoids *vs* non-exposed excluding one study where only patients without diabetes at baseline were included (sensitivity analysis)**

**
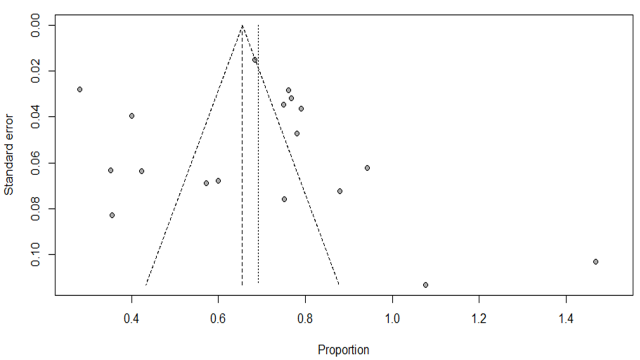
**

**Figure S2 Publication bias: prevalence of glucocorticoid-induced hyperglycaemia**

Egger’s test for publication bias: p=0.689


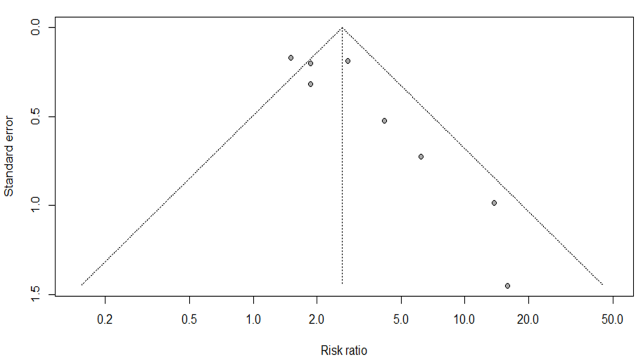


**Figure S3 Publication bias: relative risk of new-onset hyperglycaemia in people exposed to systemic glucocorticoids *vs* non-exposed**

Egger’s test for publication bias: p=0.025

**
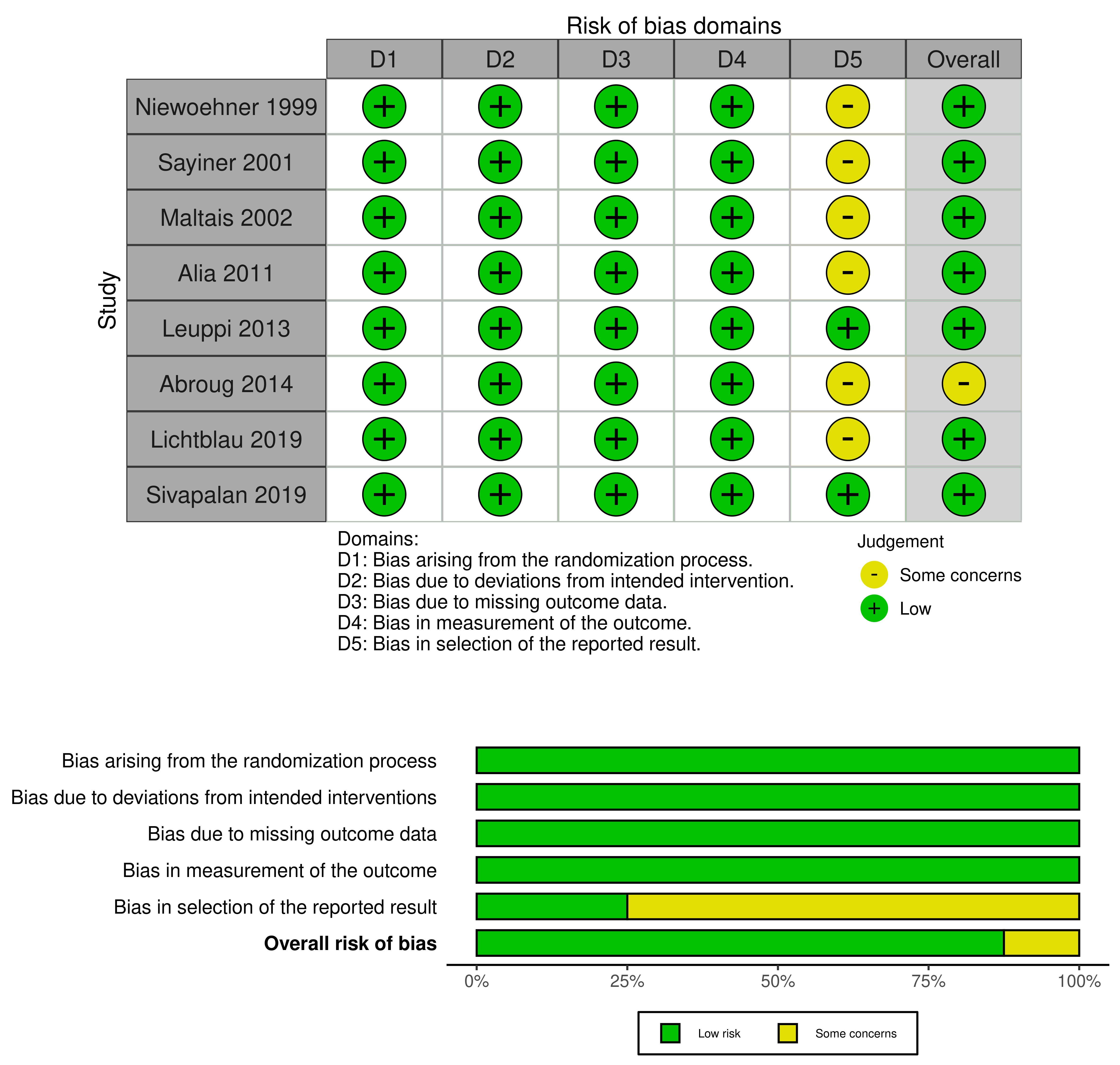
**

**Figure S4 Risk of bias assessment in randomised controlled trials using Cochrane Risk of Bias RoB2 tool**

**
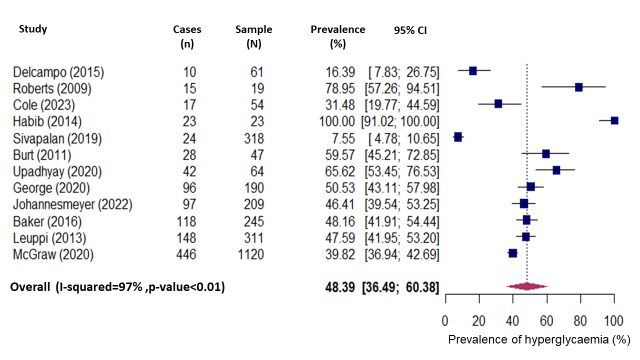
**

**Figure S5 Prevalence of glucocorticoid-induced hyperglycaemia excluding studies identified as “some concerns” in the risk of bias assessment (sensitivity analysis)**
